# An Improved EfficientNetV2 Model Based on Visual Attention Mechanism: Application to Identification of Cassava Disease

**DOI:** 10.1155/2022/1569911

**Published:** 2022-08-05

**Authors:** Yuanbo Ye, Houkui Zhou, Huimin Yu, Haoji Hu, Guangqun Zhang, Junguo Hu, Tao He

**Affiliations:** ^1^School of Mathematics and Computer Science, Zhejiang A & F University, Hangzhou 311300, China; ^2^Zhejiang Provincial Key Laboratory of Forestry Intelligent Monitoring and Information Technology, Hangzhou 311300, China; ^3^College of Information Science & Electronic Engineering, Zhejiang University, Hangzhou 310027, China; ^4^State Key Laboratory of CAD & CG, Hangzhou 310027, China

## Abstract

With the characteristic of high recognition rate and strong network robustness, convolutional neural network has now become the most mainstream method in the field of crop disease recognition. Aiming at the problems with insufficient numbers of labeled samples, complex backgrounds of sample images, and difficult extraction of useful feature information, a novel algorithm is proposed in this study based on attention mechanisms and convolutional neural networks for cassava leaf recognition. Specifically, a combined data augmentation strategy for datasets is used to prevent single distribution of image datasets, and then the PDRNet (plant disease recognition network) combining channel attention mechanism and spatial attention mechanism is proposed. The algorithm is designed as follows. Firstly, an attention module embedded in the network layer is deployed to establish remote dependence on each feature layer, strengthen the key feature information, and suppress the interference feature information, such as background noise. Secondly, a stochastic depth learning strategy is formulated to accelerate the training and inference of the network. And finally, a transfer learning method is adopted to load the pretrained weights into the model proposed in this study, with the recognition accuracy of the model enhanced by means of detailed parameter adjustments and dynamic changes in the learning rate. A large number of comparative experiments demonstrate that the proposed algorithm can deliver a recognition accuracy of 99.56% on the cassava disease image dataset, reaching the state-of-the-art level among CNN-based methods in terms of accuracy.

## 1. Introduction

Crops provide the source of human clothing and food and form the primary condition for all survivals. However, as the world is affected by the COVID-19 epidemic, labor shortages, and the flood of diseases, insect pests have led to a sharp reduction in agricultural production, setting off a wave of food shortages around the world. Among them, viral diseases are the main reason for the reduction of crop harvests. In view of the shortage of labor, the difficulty of manual detection, the variety of plant diseases, and the low identification accuracy, an efficient plant disease identification system is extremely important. As one of the three major potato crops, cassava is the third largest food crop in hot regions and the sixth largest food crop in the world. However, the current cassava production is still far from meeting the food needs of Africa and other places, and millions of Africans suffer from hunger. Cassava is the main food in Africa. During the growth of cassava, it is very meaningful to African people to quickly and accurately detect the incidence of cassava and take corresponding countermeasures, so as to improve the survival rate of cassava and increase the production of cassava.

The convolutional neural network can well complete the function of plant lesion recognition, because it can directly learn the advanced robust features of diseases from original images, rather than from manually selected or extracted features. By extracting the features of images through self-learning, CNN can greatly increase the accuracy of recognition. The input of the convolutional neural network is to package the input image and convert it into a feature matrix of the corresponding dimension, and then perform convolution, pooling, and other operations on the feature matrix to obtain the recognition accuracy of a certain category. First, CNN models often require a large amount of data for training, so as to extract useful feature information from images. However, the dataset used in this study has only a small number of samples for each disease. Second, the most samplings of the dataset used in this study are obtained by manual field shooting, and the background information of some images far exceeds the main information of the diseased leaves. Therefore, it is difficult for CNNs to learn the lesion information in complex backgrounds during training. Third, the better the CNN, the deeper the layers of the CNN, and the deeper the model will require more computing resources and a better structure to ensure the stability of the network model. Fourth, this study uses transfer learning to train the networks; accordingly, it is a difficult task to design an optimal CNN structure for identifying cassava leaf diseases.

The innovation of this study lies in applying an improved EfficientNetV2 model to identify cassava leaf lesions. The main contributions of this study are summarized as follows:

1. In order to enhance the robustness of the model and solve the problem of insufficient dataset, the images in the entire dataset are carefully selected, and any wrong information with them is corrected. In addition, to address the overfitting problem of the model, this study designs a comprehensive data augmentation method for image flipping, image mirroring, contrast and brightness changing, noise addition, image rotation, and so on. In the data augmentation strategy, the effect of cassava leaves photographed under abnormal conditions can be improved, and the augmented dataset can enhance the generalization ability of the model. Finally, a 7.7% accuracy improvement is got after augmentation in PDRNet.
2. An EfficientNetV2 model is offered for diagnosing cassava leaf diseases. For the case that the information of the diseased leaves is not remarkable in the complex background, channel attention mechanism and spatial attention mechanism are introduced in the MBConv structure to weight the subject information that the main feature information can be learned in the network training process, so as to raise the image recognition rate of the model in complex backgrounds. Meanwhile, a mixed residual connection structure is proposed on the MBConv structure to connect two attention modules to improve the stability of the model during training and the efficiency of the model. A depth-wise separable convolutional layer is applied to the PDRNet model in order to solve the problem of computational overhead in the CNN. Compared with EfficientNetV2's 98.53% recognition accuracy, the improved PDRNet shows an increase of 0.79%, reaching 99.32%.
3. A network parameter adjustment strategy is formulated. First, a strategy is adopted for dynamic adjustment to the learning rate in the process of network model learning; second, strategies for using the gradient with a momentum optimizer (SGDM) and an adaptive optimizer (Adam) are applied in the learning of the proposed network. In view of the advantages and disadvantages of SGDM and Adam, a better optimization strategy and a learning rate change strategy are adopted in the training process, so as to facilitate the network model to converge faster and deliver higher recognition accuracy. After fine-tuning, the original accuracy has increased by 0.24%, reaching 99.56%.

## 2. Related Work

In the field of agriculture, traditional machine learning methods and convolutional neural network methods are widely used for detecting plant leaf diseases. Ramcharam et al. [1] proposed that image recognition using convolutional neural network transfer learning InceptionV3 is a powerful method for high-precision automated cassava disease detection. The best model achieved an overall accuracy of 93%. Emuoyibofarhe et al. [2] developed and trained machine learning models for cassava disease detection and classification. Through the developed cubic support vector machine (CSVM) model, and the method of 5-fold cross-validation, the model has an accuracy of 83.9% in predicting the health and unhealthy status of cassava. Inspired by the hierarchical structure of taxonomic tree, Wu et al. [3] proposed the taxonomic loss. By simple group and sum operation, the hierarchical relationships among multilevel labels were encoded into the deep learning objective function. Finally, on the PlantCLEF 2017 dataset with 10,000 species, the SENet-154 model trained with the classification loss achieves 84.07%, 79.97%, and 73.61% accuracy at the family, genus, and species levels, and the model improved by 2.23%, 1.34%, and 1.08%, respectively. Sun et al. [4] established a multiple linear regression model, and the images from the training library are put into the multiple linear regression model. Then, the disease identification system is constructed by the least squares method. Through experiments, the results of the multiple regression system can better distinguish the severity of plant diseases and obtain an accuracy rate of 90% for plant disease identification under random situation. Kundu et al. [5] proposed the framework “Automatic and Intelligent Data Collector and Classifier” (AIDCC) and “Custom-Net” for automating the collection of imagery and parametric datasets from the pearl millet farmland, feature visualization, and prediction of blast and rust disease. Comparing with the state-of-the-art models, the “Custom-Net” model reports a classification accuracy of 98.78% and reduces the training time by 86.67%. By combining multiple loss functions from state-of-the-art deep CNN architectures, Dat et al. [6] conducted research on leaf image recognition. Firstly, the U-Net model was applied to segment leaf images from the background to improve the performance of the recognition system. Then, a multimodel approach based on a combination of loss functions from the EfficientNet and MobileNet (called as multimodel CNN (MMCNN)) to generalize a multiloss function was introduced. Finally, a recognition accuracy of 98.89% is got on Vietnamese herbal dataset. Wieczorek et al. [7] proposed a lightweight convolutional neural network model of face detection in risk situations to serve for faster detection of survivors, and the model detects human faces over various textures with accuracy above 99%. This model can be easily deployed to the mobile devices for using, and the continuous training model of the external server is deployed; as a result with each new confirmed face classification, the system is retrained. Therefore, the more the system is used in rescue action, the more efficient it becomes. Smart IoT infrastructure is being used to connect more mobile devices, sharing detection results between devices to more effectively coordinate rescue operations. Konstantinos [8] used AlexNet, AlexNetOWTBn, GoogLeNet, OverFeat, and VGG networks to train a model and detected 58 different types of plant and disease combinations, delivering 99.53% accuracy rate. Too et al. [9] used VGG16, ResNet50, 101, 152, InceptionV4, and DenseNet networks on the PlantVillage dataset. In the transfer learning method, after fine-tuning the network model, accuracy rates of 81.92%, 99.59%, 99.66%, 99.59%, 98.08%, and 99.75% were acquired for the above networks, respectively. Arnal and Jayme [10] adopted part of images containing diseased areas for disease identification, greatly narrowing down the data for deep learning. This method could identify different disease species on a leaf; however, it required manual segmentation of the image lesions. Compared with the original image model, the method boosted the accuracy by 12%. Based on the convolutional neural network, Anitha and Saranya [11] proposed a self-designed CNN model to train and identify cassava diseased leaves. The entire dataset is divided into 5 classes: CBB, CBSD, CGM, CMD, and Health. By performing a series of data amplification methods on the original data, the recognition accuracy rate of the model on the cassava dataset after data amplification reaches 90%. Lilhore et al. [12] developed an ECNN model to predict disease classes for a highly imbalanced cassava leaf dataset. The entire dataset is divided into 5 classes: CBB, CBSD, CGM, CMD, and Health. A depth-wise separable convolutional layer is applied to the ECNN model in order to solve the problem of computational overhead in the CNN. At the same time, the model utilizes a unique block processing feature to process unbalanced images. The use of gamma correction features in the ECNN model is proposed for the color segregation problem. In the ECNN model, the global average election polling with batch normalization is adopted to reduce the variable selection process and improve the computational efficiency. Finally, the proposed ECNN model achieved 99.3% accuracy on the balanced cassava diseased leaves dataset classifier significantly.

## 3. Materials and Methods

### 3.1. Dataset

The experimental data come from a dataset of cassava leaves taken from Uganda. The entire dataset is divided into 5 classes: Cassava Bacterial Bright (CBB), Cassava Brown Streak Disease (CBSD), Cassava Green Mottle (CGM), Cassava Mosaic Disease (CMD), and Healthy. The number of images per class is 1087, 2189, 2386, 13158, and 2577, respectively. In the experiment, the dataset is divided into 3 parts, training set, validation set, and test set. The division ratio is 7 : 2 : 1. The entire dataset has a total of 21,397 samples, with a resolution of 800 × 600.

### 3.2. Experimental Roadmap

**Figure 1:**
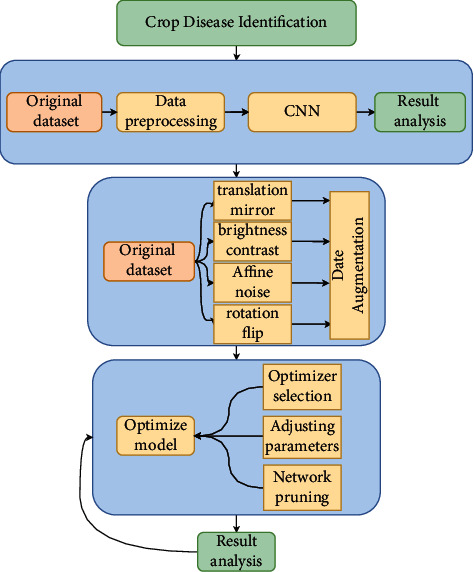
The technical roadmap. The process is to test the original data first, and then analyze and improve the experimental results by data augmentation, model optimization, parameter tuning, and other methods on the basis of the original dataset.

Figure 1 shows the technology roadmap of this study [13]. First, through general testing and analysis of the original dataset, different data augmentation strategies are carried out for the case of a small number of datasets. Then, random augmentation is performed on a picture in methods such as image translation, flipping, mirroring, rotation, noise adding, contrast adjustment, and brightness adjustment. Later on, the results of different network models are analyzed to find out the best model for this dataset. Finally, the model is optimized with a series of fine-tuning methods, such as using an optimizer and setting up learning rates, so as to further improve the recognition accuracy rate on the network model.

### 3.3. Optimizer

In the network training process, each forward pass will get the loss of the output value and the true value. The smaller the loss value, the better the model. Therefore, a gradient descent algorithm is adopted in this study to help find the minimum loss value, so as to deduce the corresponding learning parameter weights and biases and optimize the model.

#### 3.3.1. SGDM (Stochastic Gradient Descent with Momentum Algorithm)

SGDM is an algorithm for adding momentum based on the SGD optimization algorithm. The algorithm includes a learning rate *ϵ*, momentum coefficient *μ*, and the current model parameters *θ*. The initialization *v* (the accumulated acceleration at the current moment) value is 0. *g* is the current gradient. Sampling *m* sample is {*x*^(1)^,…, *x*^(*m*)^}, and corresponding target function is {*y*^(1)^,…, *y*^(*m*)^}.

Calculate gradient:

(1) $$\begin{array}{c} g\leftarrow \frac{1}{m}\nabla_{\theta }\sum_{i}L\left(f\left(x^{\left(i\right)};\theta \right),y^{\left(i\right)}\right). \end{array}$$

Update *v*:

(2) $$\begin{array}{c} v=\mu v+\left(1-\mu \right)g. \end{array}$$

Update *θ*:

(3) $$\begin{array}{c} \theta \leftarrow \theta -\epsilon v. \end{array}$$

Among them, the value of the *μ* ranges from 0 to 1. SGDM is iteratively updated once for each sample. For a large sample size, only some of the samples may be used to iterate to find the optimal solution. However, too frequent updates of SGDM may cause shocks in losses.

#### 3.3.2. Adam (Adaptive Moment Estimation) [7]

This algorithm is purposed to calculate the adaptive learning rate for each parameter. While storing the exponentially decaying average of the squared *v*_*t*_ of the past gradients, just as in AdaDelta and RMSProp algorithms, it also saves the exponentially decaying mean of the past gradients *m*_*t*_ by using the impulse method:

(4) $$\begin{array}{c} m_{t}=\beta_{1}m_{t-1}+\left(1-\beta_{1}\right)g_{t},\\ v_{t}=\beta_{2}v_{t-1}+\left(1-\beta_{2}\right)g_{t}^{2}, \end{array}$$

where *β*_1_ and *β*_2_ are two constants to be set up and *g* is the current gradient value of the error function. By default,  *β*_1_=0.9, and *β*_2_=0.999.*m*_*t*_ is 1^st^ moment vector. *v*_*t*_ is 2^st^ moment vector.

The magnitude of these two parameters indicates the relationship between the gradient update and the current and historical gradients. The larger the magnitude, the stronger the correlation between the current gradient update and the historical gradient.

If *m*_*t*_ and *v*_*t*_ are initially set to 0 vectors when they are updated for the first time, they will tend to become biased toward 0. Therefore, bias correction is necessary. *β*_1_^*t*^ and *β*_2_^*t*^ are bias correction factors. The biases can be eliminated by calculating the bias-corrected *v*_*t*_ and *m*_*t*_:

(5) $$\begin{array}{c} \hat{m_{t}}=\frac{m_{t}}{1-\beta_{1}^{t}},\\ \hat{v_{t}}=\frac{v_{t}}{1-\beta_{2}^{t}}. \end{array}$$

The algorithm's gradient update rule is

(6) $$\begin{array}{c} \omega_{t+1}=\omega_{t}-\frac{\eta }{\sqrt{\hat{v_{t}}}+\epsilon }\hat{m_{t}}, \end{array}$$

where *ϵ* indicates a constant and *η* is a learning rate (in our case 0.00001), used to prevent the denominator from becoming zero. *ω*_*t*+1_ is the model parameters at time *t* + 1.

### 3.4. Data Augmentation

**Figure 2:**
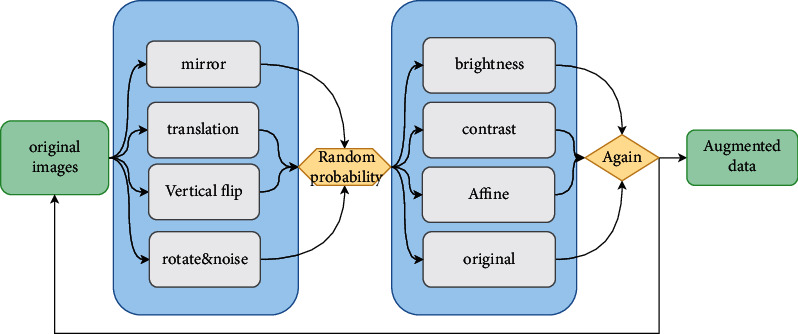
The original dataset uses a combination of digital image technology strategies for image augmentation, so as to generate different images randomly. Meanwhile, the number distribution in each category is also consistent with the original dataset.

Figure 2 illustrates the methods and steps for data augmentation [14]. In order to obtain a higher accuracy rate, it is necessary to increase the sample size of the dataset, enhance the network model, and fine-tune the hyperparameters of the network model. For a dataset obtained from any public dataset, data augmentation is an essential task. At present, data augmentation can be made in two approaches: online and offline data augmentation. For online augmentation, in the process of network model training, data are packaged into a vector form for transmission in the network, while mirroring, flipping, translating, shearing, and affine transformation are conducted on a vector [15]. However, if too many input data augmentation methods are adopted in the training process, the memory of the GPU will be greatly consumed, and the training time of the network model will also increase dramatically. Therefore, offline data augmentation is adopted. In this way, the computational burden will be diminished, and not huge time will be wasted in data preprocessing.  Table 1 shows the result of data sample augmentation.

In offline augmentation [16], operations such as image mirroring, flipping, translation, shearing, affine transformation, rotation, and noise addition are all used as augmentation methods for datasets. This dataset is divided into 5 categories, and the data distribution of each category is not uniform. Therefore, in data augmentation, that the proportion of each category is different shall be considered. Finally, through a series of augmentation processing, the original 21,397 images are expanded to 136,722 in this experiment.

#### 3.4.1. Gaussian Noise

In the next step, in order to deliver the robust generalization of image datasets, noise conforming to the Gaussian distribution is introduced in the data augmentation process. To ensure the visibility of random noise, the generated random number is multiplied by a regularization constant. The mean parameter is randomly generated from 0 to 0.5, and the deviation parameter from 0 to 0.5. The Gaussian distribution's random value generated by the random number is superimposed on the pixel value of the original image, and then it is quantized within 0∼255 to generate a Gaussian noised image.

#### 3.4.2. Gaussian Blur

The next data enhancement process implemented involves Gaussian blur, which usually refers to the lack of auto focus function in data sample collection. The blur parameters used for image transformation also follow their Gaussian distribution. This Gaussian blur is realized by the mean difference of 3, and the standard deviation is a random number among {1, 2, 3, 4, 5}.

#### 3.4.3. Rotation

Observation of the dataset shows that because of different shooting angles, the positions of the leaves in an image are usually not uniform. Therefore, in the next data augmentation, the changes of the angles are made in the image processing. And such angle changes are randomly made from 0° to 90°.

#### 3.4.4. Brightness and Contrast

It is not difficult to find in the data that the brightness and contrast information varies among different images: leaf information in some images shows high light intensity and high brightness, while other images demonstrate low brightness and relatively low contrast. In order to enhance the reliability of the dataset, it is enriched by adding brightness and contrast to some samples. Contrast changes are randomly generated with contrast parameters {1, 2.5} and brightness among {0, 1, 2, 3, 4, 5}.

#### 3.4.5. Image Translation

In order to enhance the richness of the dataset samples, an image translation operation is implemented on the dataset used in this study. Image translation is to obtain a new image by moving all the points on an image in the specified horizontal and vertical directions according to the specified translation scale. Each point on the translated image can be found in the original image.

#### 3.4.6. Mirror Image Flipping

There are two ways for mirror image flipping: vertical flipping and horizontal flipping. The former is to swap the upper half and the lower half of an image around its horizontal center axis. Similarly, the latter is to swap the left half of an image with its right half around its vertical axis.

#### 3.4.7. Bilinear Interpolation

After an image is rotated, the rotated pixel positions will be a noninteger, and holes will appear. Therefore, such holes shall be filled up by using the bilinear interpolation method and interpolate between four adjacent pixels in the image. The interpolation formula is

(7) $$\begin{array}{c} f\left(P\right)=\left(1-u\right)\left(1-v\right)f\left(i,j\right)+u\left(1-v\right)f\left(i+1,j\right)+\left(1-u\right)\text{vf}\left(i,j+1\right)+\text{uvf}\left(i+1,j+1\right). \end{array}$$

Point *P* is the coordinate that needs to be interpolated, and *u*, v are the distance difference between the coordinate position of *P* and any adjacent point. [*f*(*i*, *j*), *f*(*i*+1, *j*), (*i*, *j*+1), *f*(*i*+1, *j*+1)] are the value of 4 adjacent pixels on the image. From equation (7), the interpolated value in arbitrary coordinates can be calculated.

### 3.5. Attention Mechanism

Most images shot in the natural environment are with different backgrounds. The lesion part in some data is relatively small compared to the whole image, and the background information in some images may interfere heavily with the main information, thus causing the models to fail in learning key information or leading to an insignificant learning effect on key information. Therefore, to facilitate deep network models to learn more complex image feature information on images, the data cannot be blindly augmented [17].

The attention mechanism can deliver learning tasks in complex backgrounds to a certain extent [18]. In any deep neural network, its core calculation is made by the convolution operator, which uses the convolution kernel to learn new feature maps from the inputted feature maps. Essentially, convolution is for feature fusion of a local area, including spatial (H and W dimensions) and inter-channel (C dimension) feature fusions. For convolution operations in deep neural networks, most of the tasks are to improve the receptive field, i.e., to spatially fuse more feature information or to extract multi-scale spatial information. In ResNet [19] and GoogLeNet [20] networks, the convolution operation would basically fuse all channels of the inputted feature map by default. However, in different images, the importance of different channel feature information is distinct as well.

**Figure 3:**
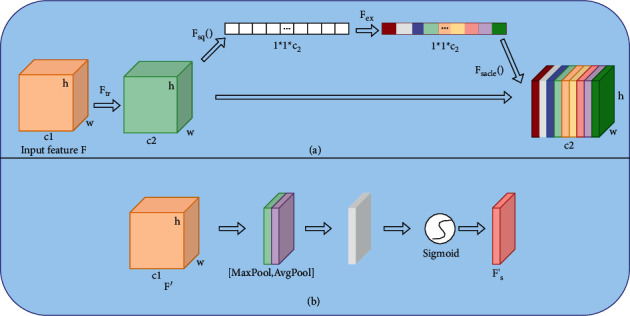
Squeeze-and-excitation module and spatial attention module. (a) Squeeze-and-Excitation Module (b) Spatial Attention Module.

Figure 3 shows the structural flowchart of squeeze-and-excitation mechanism (SE) [21] and the spatial attention mechanism (SA). SE module contains four operations: characteristic graph convolution, squeeze (*F*_sq_), excitation (*F*_ex_), and scale (*F*_sacle_).

The first step is convolution. *F*_tr_ is a simple convolution operation on an inputted feature layer, with the following formula:

(8) $$\begin{array}{c} F_{\text{tr}}:X⟶U,X\in R^{H^{\prime }*W^{\prime }*C^{\prime }},U\in R^{H*W*C},\\ u_{c}=v_{c}*X=\sum_{s=1}^{c}v_{c}^{s}*x^{s}. \end{array}$$

The convolution kernel is *V*=[*v*_1_, *v*_2_,…, *v*_*c*_], where *v*_*c*_ represents the *c*-th convolution kernel and *X* represents input feature vector. Output: *U*=[*u*_1_, *u*_2_,…, *u*_*c*_] represents output feature vector.

The second step is for the squeeze operation. Since convolution operates only in a local space, it is difficult for *U* to obtain enough information to extract the relationships between different channels. Therefore, the squeeze operation encodes the entire spatial feature on a channel as a global feature and then uses a global average pooling operation to get the results. The formula is

(9) $$\begin{array}{c} Z_{c}=F_{\text{sq}}\left(u_{c}\right)=\frac{1}{H*W}\sum_{i=1}^{H}\sum_{j=1}^{W}u_{c}\left(i,j\right),\;Z\in R^{C}. \end{array}$$

In the third step, the excitation operation uses the global description features obtained from the squeeze operation. And then, it is necessary to find out the relationship between different channels. This operation needs to meet two criteria: first, it must be flexible enough to learn the nonlinear relationships between all channels; second, the learned relationships cannot be mutually exclusive. Because the features of all channels are needed, not in one-hot form, the following formula of the sigmoid activation function is used to quantify the outputted weight coefficient of the last layer from 0 to 1:

(10) $$\begin{array}{c} s=F_{ex}\left(Z,W\right)=\sigma \left(W_{2}\text{ ReLU}\left(W_{1}Z\right)\right), \end{array}$$

where *W*_1_ ∈ *R*^*C∗*(*C*/*r*)^,  and *W*_2_ ∈ *R*^*C∗*(*C*/*r*)^. *W*_1_ and *W*_2_ represent two fully connected layers with different dimensions. In order to reduce the complexity of the model and improve the generalization ability, a bottleneck structure containing two full connection layers is adopted. The first full connection layer plays the role of dimension reduction, with the super-parameter *r* acting as the dimension reduction coefficient, and then the ReLU activation function is implemented. The second full connection layer would restore the feature dimension to the original input dimension.

The last step is the scale operation: multiply the learned activation value of each channel (after sigmoid activation, the value is 0∼1) with the original feature *U* by using the following formula:

(11) $$\begin{array}{c} F_{\text{scale}}\left(u_{c},s_{c}\right)=s_{c}*u_{c}. \end{array}$$

The channel attention mechanism mainly produces effects on the channel, so that the network model understands what part of the feature map should have a higher response. However, the network model is still not clear about where the feature map has a higher response. Spatial attention mechanism can effectively solve this problem. Applying a pooling operation along the channel axis effectively highlights informative regions. To compute the spatial attention, *F*_avg_′ ∈ *R*^1×*H*×*W*^ and *F*_max_′ ∈ *R*^1×*H*×*W*^ the two 2D maps were generated by aggregating the channel information of the feature maps using two average pooling and max pooling. Those then were concatenated and convolved through a convolution *l* layers to generate a spatial attention map *F*_*s*_′ ∈ *R*^*H∗W*^ which encodes where to emphasize or suppress [22]. The spatial attention is computed as

(12) $$\begin{array}{c} F_{s}^{\prime }=\sigma \left(f^{7*7}\left(\left[F_{\text{avg}}^{\prime };F_{\text{max}}^{\prime }\right]\right)\right), \end{array}$$

where *σ* is the sigmoid activation function and *f*^7*∗*7^ is a convolutional layer with a kernel size of 7 ∗ 7.

### 3.6. Network Model

**Figure 4:**
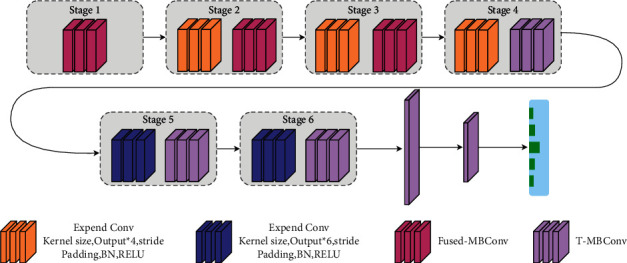
The structure of the PDRNet network.

This experiment uses the EfficientNetV2 network, which has introduced the attention mechanism module. On this basis, PDRNet network is proposed. Compared with EfficientNetV2, PDRNet introduces a stochastic depth and a spatial attention mechanism. The stochastic depth is added to speed up the initial training of the network and the network inference. The spatial attention mechanism enables the network model to learn where on the feature map there is a higher response. The PDRNet uses a strategy combining Fused-MBConv convolution and T-MBConv convolution in its structures, with the Fused-MBConv and T-MBConv structures shown in Figure 4. The shallow layer of the PDRNet network adopts Fused-MBConv, while the deep network layer adopts the T-MBConv convolutional structure. Then, a dropout layer is inserted in both the Fused-MBConv and T-MBConv structures, while taking effect with only shortcut branches. The role of this layer is to randomly discard the main branch and connect it to the next layer through a shortcut branch, delivering the effect of randomly reducing the network levels, so as to slash the training time in the training process and significantly boost the inference speed.  Table 2 makes a comparison between using stochastic deep learning. Performance improved by 28.57%.

In the experiment, we redefine part of the layer structure in the efficient network; in stage4 to stage6, the spatial attention (SA) module is embedded in the stage. The SA module is directly embedded behind the SE module by splicing to form the SE-SA structure. Through those changes, PDRNet was proposed.  Table 3 illustrates the details of each module at the network level of the PDRNet network. Figure 4 shows the network structure of the PDRNet.

**Figure 5:**
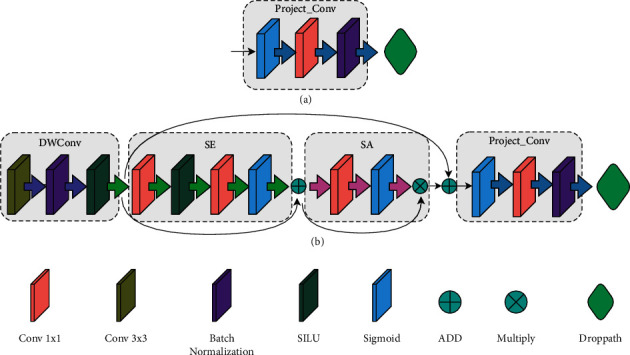
Method of mixed residual connection. (a) Fused-MBConv. (b) T-MBConv.

Figure 5 shows the attention mechanism mixed residual connection method used in this paper, where *a* and *b* are the convolutional structures of Fused-MBConv and T-MBConv. In the experiment, the attention module in T-MBConv is improved. T-MBConv is a modified structure based on the MbConv. The difference from the original MBConv is that the spatial attention mechanism is embedded, and the connection of each module in the T-MBConv uses a mixed residual connection structure. While introducing spatial attention mechanism, a mixed residual connection method is used to splicing it into the network backbone. After the feature layer passes through the SE module, it is added to the original feature layer, so that the network can learn the information concerned by the attention module during the training process and will not lose the original underlying information as the network level increases. Then, the output of the SE module passes through the spatial attention (SA) module, and the output containing the spatial information is multiplied by the output of the SE module to obtain a new feature layer. In this way, the feature layer contains the information of what the object is. It also contains information about where the object is. In the same way, in order to prevent the overfitting of the model during the convergence process due to too much attention to spatial information in the deep network, we add the original features input to the T-MBConv module and the output of the SA module. As shown in Figure 5, the outputs of the SE and SA modules are multiplied, and when we expand the model capacity, we observe a significant increase in activations at deeper layers. In fact, in the normalization configuration, the value of each residual block is directly merged into the main branch, the amplitude of the main branch is larger than that in the deeper layers, and the large difference between different layers leads to unstable training. In order to alleviate this problem, we use the residual normalization method, as shown in *b* in Figure 5. In this method, the output of each residual block is normalized before being merged into the main branch, and the amplitude of the main branch does not accumulate as the layer goes deeper. At the same time, the first module in Project-Conv uses a normalization operation again to alleviate the problem that the output amplitude is too different from the main branch after multiplying the output of SE and the output of SA. The activation amplitudes by this approach are much milder than in the original pre-normalization configuration.

### 3.7. Fine-Tuning

In order to optimize the fitting ability of the network model to the research dataset, this study makes slight adjustments to the EfficientNetV2 [23] network model by replacing the last layer of the network with two fully connected layers containing ReLU activation function. On this basis, a softmax layer and a fully connected layer with node five are added. The last layer of the network is deleted and connected with two fully connected layers. The ReLU activation function is used in the middle of the two fully connected layers. The last layer is a fully connected layer and a softmax layer with 5 nodes.

The influence of the settings of the hyperparameters in the model on the network is non-negligible. The settings of hyperparameters corresponding to different optimization methods for the same network vary greatly. Both Adam optimizer and SGDM optimizer are adopted in this study, and the corresponding network hyperparameters are set to them.

Both optimizers have their own advantages and disadvantages [24]. Adam optimizer can quickly converge by adopting adaptive learning rate in the initial training of the network. However, the rapid attenuation of learning rate will prevent Adam optimizer from updating network parameters in the later stage of network training, thus leading to non-convergence of the network model and the inability to achieve the global optimum. The SGDM optimizer depends very much on the selection of the learning rate in the process of network training. Proper parameter settings would help the model reach the global optimum in a shorter period of time. If the learning rate is set to a too high value, the network will miss the global optimum or vibrate near the global optimum. However, if the learning rate is set to a too small value, the convergence of the network will become too slow and impossible. Therefore, at the initial stage of network training, Adam optimization strategy would be used first at a learning rate of 0.001. When the network is trained quickly and reaches the global optimum, SGDM optimizer would be used to set a smaller learning speed, so as to make the network converge slowly to the global optimum of the model.

**Figure 6:**
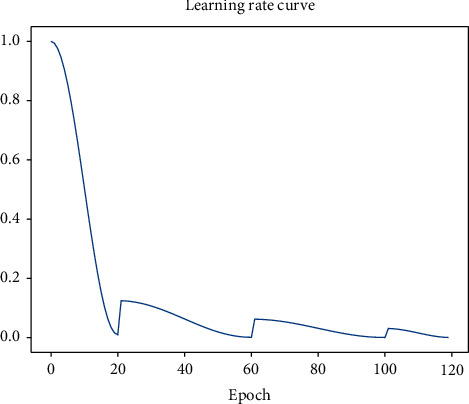
The dynamic change curve of the learning rate of the model drawn by the pseudo-code, the cosine annealing mode is used to decay at 0∼120 epoch, and the value of the learning rate is adjusted to 1/8, 1/16, 1/32 at the 20th, 60th, and 100th epoch.

In the late stage of the network model training, the network may be trapped in a local optimal point during the convergence process, and even the optimizer with momentum cannot jump out of the local optimal value. As a result, the network model will oscillate around the local optimal point for ever. Algorithm 1 is the implementation step of this dynamic learning rate. *θ*_*t*_ is the current epoch value, and *f*(*θ*_*t*_) is the curve formula. *P* is the curve corresponding to *f*(*θ*_*t*_).  Figure 6 shows the curve of dynamic learning rate. When the network loss is fluctuating greatly, appropriately increasing the learning rate will help the network jump out of the local optimum to continue its learning.

## 4. Results

### 4.1. Experimental Environment

In this experiment, the cassava leaf disease identification model only uses one computer device in the whole training and validation process. The training of the CNN operates in the graphics processing unit (GPU) mode. The detailed characteristics of the computer used in this experiment are described in Table 4.

### 4.2. Effectiveness Experiment of the Module

#### 4.2.1. Effectiveness Experiment of Fine-Tuning

**Figure 7:**
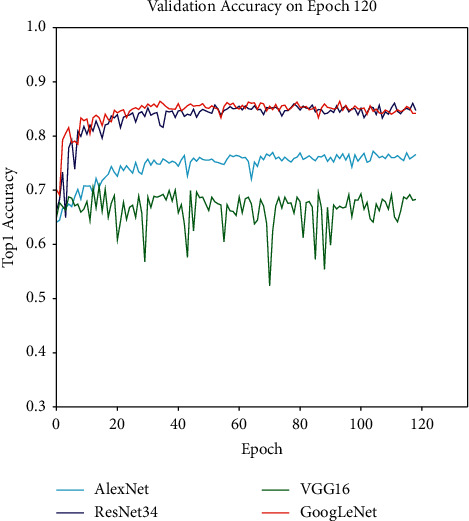
The image is the verification accuracy curve of VGG16 and GoogLeNet in the original dataset.

In all experiments, whether training or validation, the image size was resized to 224 ∗ 224, and the batch size was 16. Before data augmentation, the classic VGG16 and GoogLeNet networks were first used to train the original dataset, so as to obtain the accuracy rate of the dataset without any processing. The network model and network parameters used are not fine-tuned. The experimental results are shown in Figure 7.

**Figure 8:**
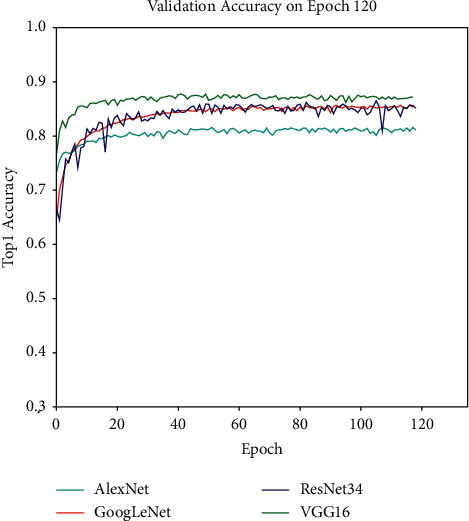
The validation accuracy rate of the network AlexNet, GoogLeNet, VGG16, and ResNet34 on the no-augmented dataset.

**Figure 9:**
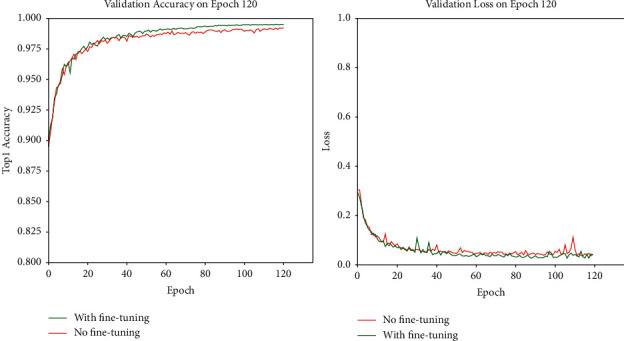
PDRNet model validation accuracy and loss curve comparison chart. The left image shows the accuracy curves of the fine-tuned and un-fine-tuned network models. The left image shows the loss curve of the model fine-tuned and not fine-tuned.

As shown in Figures 7 and 8, the accuracy rate is improved after fine-tuning. The model's training curve and loss curve are smoother than those without parameter tuning. Therefore, the hyperparameters of the network need to be adjusted before the network is trained. For instance, the learning rate can be adjusted. If the network converges too quickly and oscillates, the learning rate may have been set to a too large value, so it should be appropriately reduced to optimize the network, with the adjustment method shown in Figure 6. Then, the Adam optimizer is used at the beginning of training at a learning rate of 0.001; finally, the network model is trained with the SGDM optimizer at a learning rate of 0.0001 in the last 20 epochs. In the final experiment, we also use the same fine-tuning method to adjust the model, and the experiments show that the fine-tuning method is effective.  Figure 9 shows a comparison of the validation results before and after model fine-tuning. After dynamic adjustments to the learning rate the optimizer uses during the network training process, the verification accuracy of the model can be significantly improved. In each stage of the training, the accuracy before fine-tuning is higher than that without fine-tuning in the same stage, the loss curve becomes smoother than before, and the loss value after fine-tuning is lower than that before fine-tuning.

#### 4.2.2. Effectiveness Experiment of Augmentation Dataset

**Figure 10:**
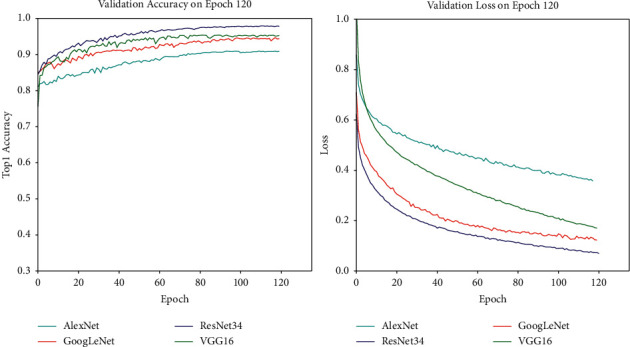
The validation accuracy rate of the network AlexNet, GoogLeNet, VGG16, and ResNet34 on the augmented dataset. The image at the left is the validation accuracy curve of each network model, and the image right is the verification loss curve of each network model.

As shown in Figure 8, the original dataset could not make the network reach a satisfactory recognition accuracy rate, because the sample dataset is not big enough to develop an advanced classification model based on deep learning. In addition, due to the existence of individual insect diseases, the appearance characteristics of leaf diseases show little difference in their external manifestations. As a result, multi-classification research on insect diseases could not be carried out with the machine learning technology of pixel-level handcrafted features.  Figure 10 indicates that the same model delivers great differences between the no-augmented dataset and the augmented dataset. The recognition accuracy rate of the model on the dataset without the operation of data augmentation is generally lower than that of the augmented dataset. For example, the maximum accuracy rate obtained by AlexNet on the original dataset is 79.7%, while that obtained on the augmented dataset after training by AlexNet network would reach 89.8%, increased by 10.1%. The experimental results of different deep network models on the dataset before and after augmentation are shown in Table 5.

#### 4.2.3. Effectiveness Experiment of the Attention Module

**Figure 11:**
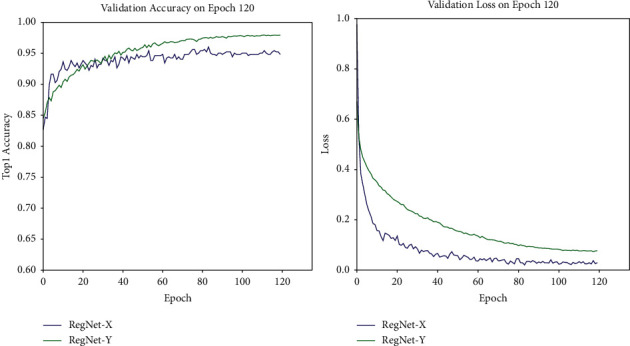
The left image is the validation accuracy curve of the RegNetX and RegNetY networks. On the right are the loss curves of the two networks.

Figure 11 compares the experimental results of the same network with and without the attention module. For some datasets, the image background is so complex that the subject feature information is not remarkable. Thus, it is necessary to add an attention mechanism during network training, so as to learn the inconspicuous part of the dataset feature information. In order to effectively find out whether the deep neural network can improve the accuracy after adding the SE module [25], two network models, RegNetX [26] and RegNetY, are implemented in the training; then, the results will be compared. No SE module is added to RegNetX, but an SE module is to RegNetY.

Table 6 shows that the verification accuracy of RegNetX without the SE module is 95.6%, and that with the SE module is 98.4%. Therefore, after the SE module is adopted, RegNetY can improve its accuracy by 2.8 percentage points. Therefore, it can be concluded that for the same dataset, the network model with the SE module would perform significantly better than that without such module in terms of validation accuracy.

#### 4.2.4. Effectiveness Experiment of the PDRNet

**Figure 12:**
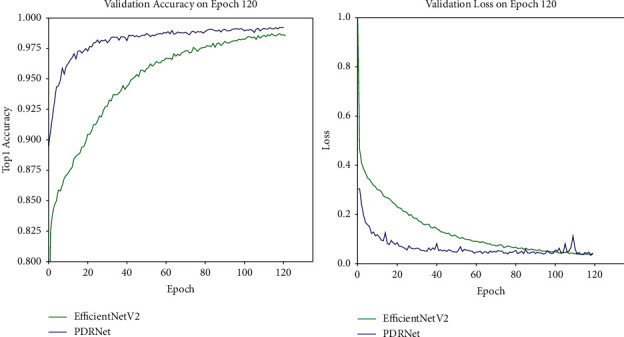
The experimental results of EfficientNetV2 and PDRNet on the augmented dataset. The left image shows the accuracy curve of EfficientNetV2 and PDRNet networks. The right image is the loss curve.

After analysis of the gap between those network models of RegNetX, RegNetY, and EfficientNetV2, the EfficientNetV2-based network is finally selected for the classification study of cassava leaf diseases. Meanwhile, the EfficientNetV2 network architecture is updated for use. Firstly, the network model lacks the learning of the spatial location information of the feature map, and the spatial attention module was embedded into the EfficientNetV2. Each of them was embedded after the SE module. The last layer of the network is replaced with two fully connected layers; the ReLU activation function is used in the middle of the two fully connected layers; the last layer is a fully connected layer with 5 nodes and a softmax layer. Finally, the new network PDRNet is proposed. Figure 5 shows the PDRNet structure used after the update.  Figure 12 presents the validation results and losses of the EfficientNetV2 and PDRNet on the dataset. The accuracy rates of the two models are shown in Table 6.

### 4.3. Comparison Experiment

To thoroughly validate the effectiveness of the method proposed in this paper, we use the same dataset and experimental environment in each experiment, changing only those that need to be compared. In order to compare which combination of attention mechanisms is optimal for this model, we use EfficientNetV2 as the backbone network and only replace the attention module in the network structure. In the experiment, we compared four different attention combination strategies as separate SE module, separate SA module, SA-SE module, and SE-SA module. We also introduce the coordinate attention (CA) mechanism in our experiments. A coordinate attention block can be viewed as a computational unit designed to enhance the expressiveness of features in mobile networks. It can take any intermediate feature tensor as input and transform the output with the same size as the tensor with enhanced representation. During the experiment, the CA module is embedded in the network instead of the original module. The final results achieved by different attention combination strategies on the same dataset are shown in Table 7.

### 4.4. Comparison with Other Networks

**Figure 13:**
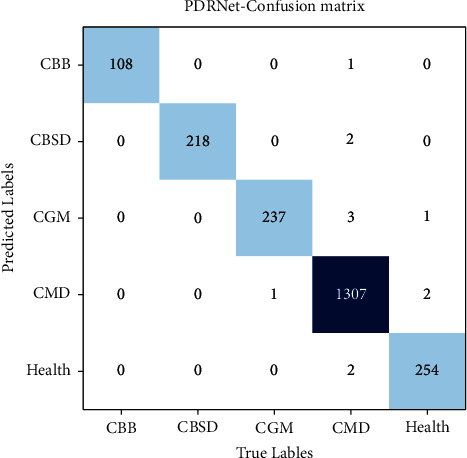
Confusion matrix results of PDRNet model, where CBB, CBSD, CGM, CMD, and Health are the abbreviations for the five categories of the dataset. The corresponding number of test images is 108, 218, 238, 1315, and 257.

Figure 13 indicates that the results of the model prove that the experimental results of the following nine models are significantly improved on the validation set: AlexNet, VGG16, GoogLeNet, ResNet34, RegNetX, RegNetY, EfficientNetV2, and PDRNet. Like the training set, the experimental set divides its samples into five categories, and the number of tests in each category is one tenth of the original dataset. The validation results of different models are presented in the form of confusion matrix [10]. Figure 13 shows the recognition results of PDRNet for each category using the confusion matrix, while showcasing the statistics on the average accuracy rate, average recall, and average specificity of each model through Table 8.

More intuitively, Table 9 showcases the differences between the network models used in the experiment, the accuracy of each network model, the verification accuracy, the model parameter flops, the inference time, and the training time. The comparison of the experiments in the task for the diseased leaves reveals that PDRNet is superior to other models in terms of verification accuracy and inference prediction speed. Moreover, it is worth noting that the dataset samples used are not large enough. If the dataset is gradually increased, the recognition accuracy of the PDRNet may be enhanced at the current inference speed.

### 4.5. Performance on the Plant Village Public Dataset

**Figure 14:**
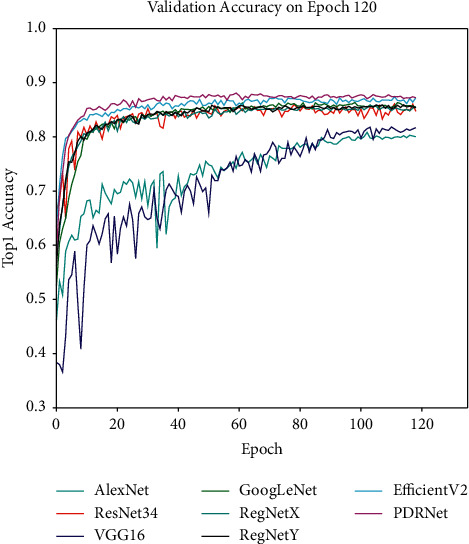
Accuracy plots for all experimental models.

In order to verify whether the network model proposed in this paper has a common effect on other datasets, we re-experiment on the PlantVillage dataset. On the PlantVillage dataset, we only selected 15 tomato lesions as the experimental data, with a total of 11148 images, and divided them into train set and validation set. The division ratio is 7 : 2 : 1. In order to compare the effectiveness of our proposed network structure, we have verified all the previous networks, and the verification results are shown in Figure 14. The statistics of the average precision, average recall, and average specificity of each model are presented in Table 10. These results show that our proposed network structure still achieves the state of the art in terms of accuracy among CNN-based methods.

## 5. Conclusion

By comparing the experimental results of different network models on the augmented and nonexpanded datasets, it is concluded that data augmentation is crucial to the improvement in the accuracy of the experimental results. For the recognition of complex images, an attention mechanism is proposed to weight different feature channels through network learning, retain or amplify important information feature channels, and suppress or discard useless information channels. Compared with the model without the attention module added, the accuracy can be raised by 2.8% with such addition. The training time and model parameters of the same model are almost the same as the original model after the attention module is implemented. In order to balance the computing time and make up for the loss in the case of time complexity, stochastic deep learning strategies are introduced. Without losing any accuracy, the model's training speed and inference speed are both boosted, a great improvement to the generalization of the model. Compared with EfficientNetV2's 98.53% recognition accuracy, the improved PDRNet shows an increase of 0.79%, reaching 99.32%. Finally, based on discussion of the selection strategy and combination strategy for different optimizers, as well as the method of lowering the learning rate and fine-tuning the model, the PDRNet is further improved and optimized to achieve a higher accuracy rate. After fine-tuning, the original accuracy has increased by 0.24%, reaching 99.56%, while the loss curve becomes smoother. However, the number of layers of the network model adopted in this study is redundant, and its slow convolution operation leads to much more network parameters and training time than other models. How to optimize the number of network layers and enhance the convolution method in the network is still an issue for the future work.

## Figures and Tables

**Algorithm 1:**
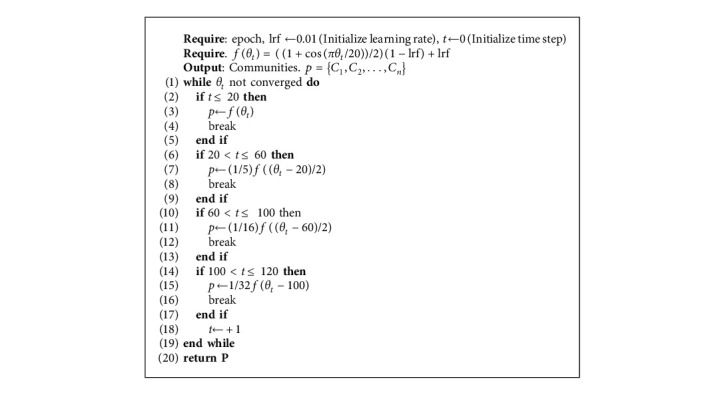
Dynamic attenuation of learning rate.

**Table 1:** Data samples of the original image and data augmentation.

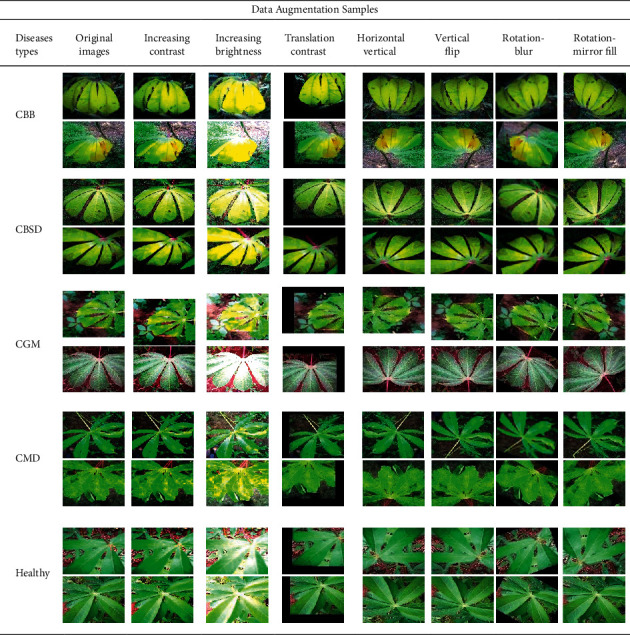

**Table 2:** The time comparison table of adding random depth and not adding random depth. The training time is the time it takes for the model to train for 120 epochs. The meaning of inference time is the time it takes for the model to predict a single image.

Model	Train time (h)	Infer time (s)
With dropout	35	1.01
No dropout	49	1.4

**Table 3:** PDRNet structure. T-MBConv and Fused-MbConv blocks are described in Figure 5.

Stage	Operator	Stride	Channels	Layers
0	Conv3 ∗ 3	2	24	1
1	Fused-MBConv1,k3 ∗ 3	1	24	2
2	Fused-MBConv4,k3 ∗ 3	2	48	4
3	Fused-MBConv4,k3 ∗ 3	2	64	4
4	T-MBConv4,3,SESA0.25	2	128	6
5	T-MBConv6,3,SESA0.25	1	160	9
6	T-MBConv6,3,SESA0.25	2	272	15
7	Conv1 ∗ 1&Pooling&FC	-	1280	1
8	Conv1 ∗ 1&RELU&FC	-	5	1

**Table 4:** Basic machine characteristics.

Hardware and software	Characteristics
Memory	11 Gb
Processor	Intel(R) Core(TM) i7-7800X CPU @ 3.50 GHz
Graphics	NVIDIA GeForce GTX 1080 Ti ×2
Operating system	Ubuntu 18.04; Python 3.8; PyTorch 1.80

**Table 5:** The validation accuracy of the different networks. All validation results are done on 1080Ti.

Method	Original dataset (%)	Augmentation dataset (%)
ResNet34	84.9	**97.8**
VGG16	**85.2**	95.3
GoogLeNet	84.1	94.9
AlexNet	79.7	89.8

**Table 6:** The accuracy of RegNetX, RegNetY, EfficientNetV2 [27], and PDRNet.

Model	Accuracy (%)
RegNetX	95.6
RegNetY	98.4
EfficientNetV2	98.53
PDRNet	**99.32**

**Table 7:** Experimental results of different combinations of attention modules.

Method	Top 1 Acc (%)
SE	86.66
SA	85.54
SA-SE	87.01
SE-SA	87.77
Coordinate attention	88.01
PDRNet-SE-SA	**88.19**

**Table 8:** Average precision, average recall, and average specificity of various models.

Model	Precision (%)	Recall (%)	Specificity (%)
AlexNet	90	90.4	97.60
VGG16	96.3	96.5	99.20
GoogLeNet	95.0	95.1	98.80
ResNet34	97.8	97.72	99.42
RegNetX	95.4	95.34	98.84
RegNetY	98.36	98.3	99.56
EfficientNetV2	98.45	99.2	99.70
PDRNet	**99.1**	**99.54**	**99.88**

**Table 9:** Different network performance results on augmentation dataset. Infer time is measured on 1080ti GPU with batch size 16 using the same codebase; train time is the total training time. All models are trained with transfer learning.

Model	Top1 Acc (%)	Param (M)	FLOPs (G)	Infer time (s)	Train time (h)
AlexNet	89.8	57.0	0.8	2.01	**4.8**
VGG16	96.3	102	15.5	1.58	22.4
GoogLeNet	94.9	10.2	1.52	1.02	7.8
ResNet34	97.8	22.3	3.68	1.306	8.7
RegNetX	95.6	5.5	0.43	1.21	25
RegNetY	98.4	**5.1**	**0.42**	1.32	27
EfficientNetV2	98.53	21.4	2.908	1.01	35
PDRNet	**99.56**	21.5	2.909	**1.01**	35

**Table 10:** Average precision, average recall, average specificity, and top 1 accuracy of various models.

Model	Precision (%)	Recall (%)	Specificity (%)	Top1 Acc (%)
AlexNet	81	81.2	89.6	80.8
VGG16	81.6	82	90.32	81.7
GoogLeNet	85.9	85.84	92.1	85.7
ResNet34	86	86.12	93.22	86.06
RegNetX	85.7	85.74	92.5	85.83
RegNetY	86.05	86	93.1	86.02
EfficientNetV2	87.36	88.2	95.2	87.4
PDRNet	**88.01**	**87.84**	**98.34**	**88.1**

## Data Availability

The data used to support the findings of this study are available from the corresponding author upon request.
